# An alternative surgical approach reduces variability following filament induction of experimental stroke in mice

**DOI:** 10.1242/dmm.029108

**Published:** 2017-07-01

**Authors:** Melissa Trotman-Lucas, Michael E. Kelly, Justyna Janus, Robert Fern, Claire L. Gibson

**Affiliations:** 1 Department of Neuroscience, Psychology and Behaviour, University of Leicester, Leicester LE1 9HN, UK; 2 Preclinical Imaging Facility, Core Biotechnology Services, University of Leicester, Leicester LE1 9HN, UK; 3 Peninsula School of Medicine and Dentistry, University of Plymouth, Plymouth PL6 8BU, UK

**Keywords:** Animal models, Brain ischemia, MRI, Transient MCAO, Carotid artery ligation

## Abstract

Animal models are essential for understanding the pathology of stroke and investigating potential treatments. However, *in vivo* stroke models are associated, particularly in mice, with high variability in lesion volume. We investigated whether a surgical refinement where reperfusion is not reliant on the Circle of Willis reduced outcome variability. Mice underwent 60 min of transient middle cerebral artery occlusion avoiding ligation of the external carotid artery. During reperfusion, the common carotid artery was either ligated (standard approach), or it was repaired to allow re-establishment of blood flow through the common carotid artery. All mice underwent MRI scanning for assessment of infarct volume, apparent diffusion coefficient and fractional anisotropy, along with terminal assessment of infarct volume by 2,3,5-triphenyltetrazolium chloride (TTC) staining. Repairing the common carotid artery following middle cerebral artery occlusion enhanced reperfusion (*P*<0.01) and reduced the variability seen in both total (histological analysis, *P*=0.008; T2-weighted MRI, *P*=0.015) and core (diffusion tensor MRI, *P*=0.043) lesion volume. Avoiding external carotid artery ligation may improve animal wellbeing, through reduced weight loss, while using an alternative surgical approach that enabled reperfusion through the common carotid artery decreased the variability in lesion volume seen within groups.

## Abstract (precis)

**Summary:** An alternative surgical approach following middle cerebral artery occlusion, which allows reperfusion through the common carotid artery, decreases the variability in lesion volume seen within groups and reduces the number of animals required to detect a treatment effect.

## INTRODUCTION

Focal ischemia in the territory of the middle cerebral artery is one of the major causes of clinical stroke. Despite numerous clinical drug trials, tissue plasminogen activator (tPA) remains the only specific pharmacological treatment with proven efficacy for ischemic stroke (O'Collins et al., 2006; Sutherland et al., 2012). Owing to its narrow therapeutic window (<4.5 h) and safety concerns, only ∼15% of stroke patients receive tPA and recanalization rates can be less than 50% (Reeves et al., 2005; Wardlaw et al., 2003). The failure of translation from experimental efficacy of potential treatments to clinical utility is a major concern for stroke (Pangalos et al., 2007), and probably relates to poor clinical trial design, delays in treatment, clinical stroke heterogeneity and possible limitations in the animal models (Hossmann, 2009). The development of safe and effective treatments remains a major challenge for stroke research.

Animal models of ischemic stroke have been developed to investigate the underlying pathophysiological mechanisms and to identify pharmacological targets. Such models produce animals with heterogeneous lesion volumes with large standard deviations, including animals with zero lesion volume even though surgical protocols were applied in accordance with a defined protocol (Carmichael, 2005; Dirnagl, 2006; Ingberg et al., 2016). Intraluminal filament occlusion of the middle cerebral artery (MCAO) in mice is the most frequently used rodent model of experimental stroke as it allows the restoration of blood flow after the induction of focal ischemia thus mimicking the sequence of events in human stroke (Ringelstein et al., 1992). In order to permit reperfusion the vessel through which the filament is inserted, which is either the common carotid artery (CCA) or the external carotid artery (ECA), remains permanently ligated (Macrae, 2011). Permanent ligation of the CCA prevents re-establishment of blood flow through the internal carotid artery and reperfusion is then wholly reliant on collateral supply within the Circle of Willis. This structure has a high anatomical variability, particularly in C57Bl/6 mice (McColl et al., 2004), which are typically used in experimental stroke studies. Although the alternative is to insert the filament via the ECA, if vascular supply to the ECA is compromised this has been shown, in rats, to have a detrimental effect on the animal's wellbeing (Trueman et al., 2011).

Reliance on the Circle of Willis for reperfusion in the established models may account, at least in part, for the variability seen in lesion volume following MCAO. In addition, filament insertion via the ECA is likely to impact on animal wellbeing. Thus, we investigated an alternative surgical approach in mice whereby ECA ligation, even temporarily, is avoided and the incision in the CCA is repaired, thus permitting reperfusion via the CCA and independent of the Circle of Willis. Repair of the CCA has been previously shown in rats to permit successful reperfusion through the CCA (Dittmar et al., 2005). We predict that in mice, wellbeing can be improved by avoiding ECA ligation and that if we can re-establish reperfusion via the CCA then variability within this model will be reduced.

## RESULTS

### Protocol changes for improved wellbeing after MCAO

**Fig. 1.**
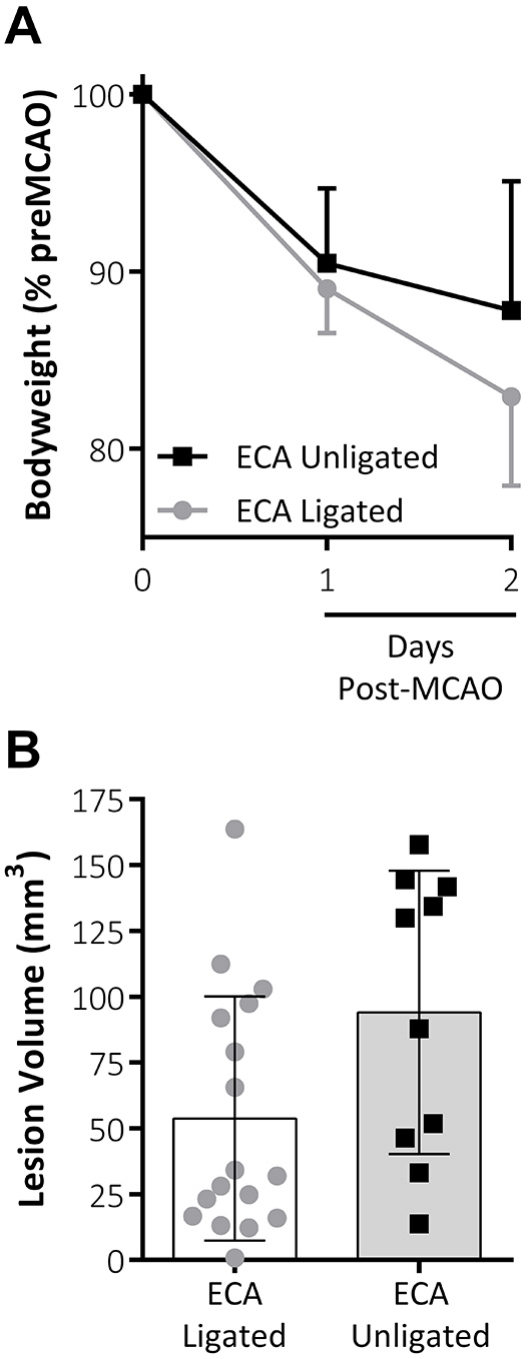
**Effect of combined analgesia treatment and omission of ECA ligation.** (A) Body weight (% of pre-MCAO body weight) is reduced at 1 and 2 days post-MCAO in both groups. There was a trend towards improved weight loss at 2 days post-MCAO following introduction of analgesia and avoiding ligation of the ECA. (B) Lesion volume (mm^3^) as measured by TTC staining at 48 h post-MCAO. Data shown are mean±s.d., ECA unligated, *n*=10; ECA ligated, *n*=17.

We performed MCAO without ECA ligation and with analgesia (‘ECA unligated’) and compared body weight and lesion volume with results in mice from previous data that had undergone the same time period of MCAO under the same surgeon but with ECA ligation and no analgesia (‘ECA ligated’). A trend towards improved body weight loss at 48 h post-MCAO within the ECA unligated group (12.19±7.29% weight loss) is shown compared with the ECA ligated group (17.05±5.03% weight loss; Fig. 1A). Avoiding ECA ligation and using an analgesic regime decreased body weight loss but did not appear to affect the total lesion volume at 48 h post-MCAO compared with results in ECA ligated animals (Fig. 1B). However, the effects of avoiding ECA ligation and introducing analgesia were not compared separately.

### Repairing the CCA to enhance reperfusion

**Fig. 2.**
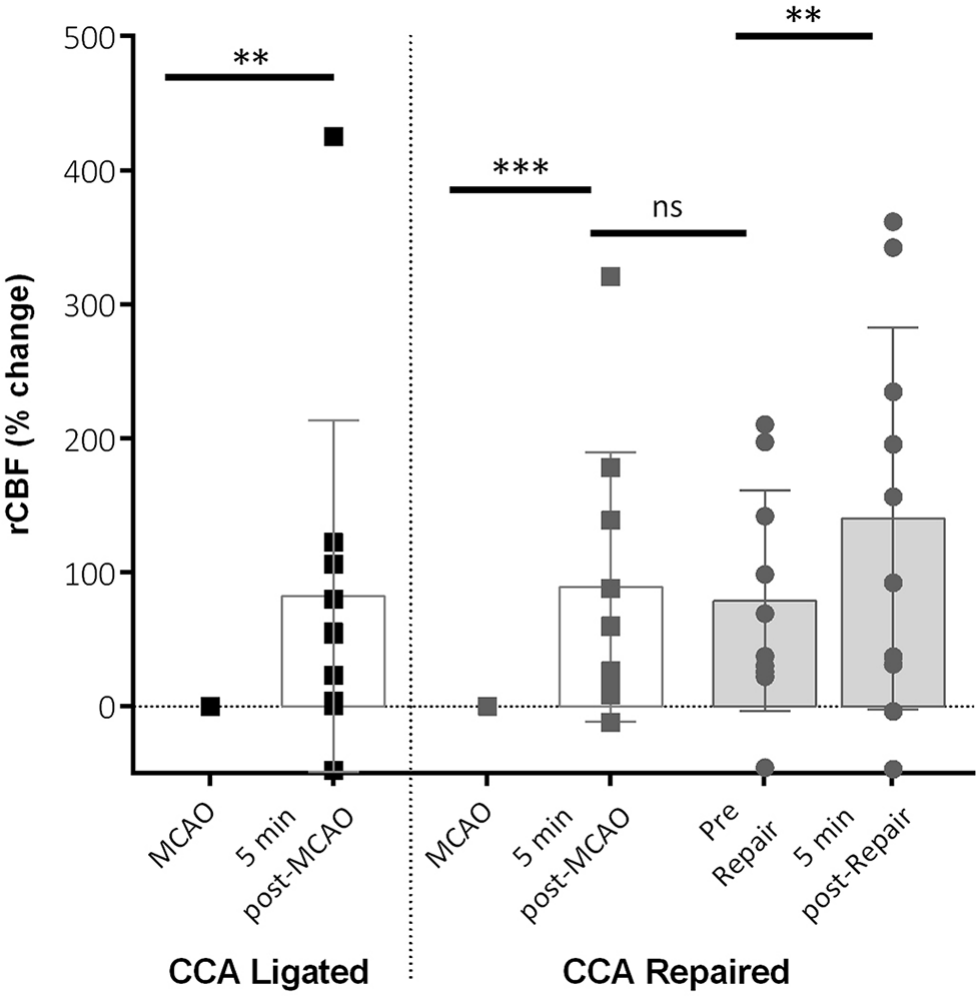
**Regional cerebral blood flow (rCBF) parameters post-MCAO.** Changes in rCBF at 5 min post-MCAO (filament removal) in both groups and in the CCA repair group. Data is shown for rCBF immediately pre-CCA vessel repair (pre-repair) and 5 min post-CCA repair. rCBF increased significantly after filament removal in both groups, with a further increase shown post-repair in the CCA repair group. No difference was seen between 5 min post-MCAO and pre-repair. Data shown are condensed from analysed time course data showing mean±s.d. for key time points. CCA ligated, *n*=10; CCA repaired, *n*=10; **P<0.01, ***P<0.001, ns, non-significant.

In the current study, we compared the effect of allowing reperfusion to be re-established through the CCA following repair with the standard approach for MCAO whereby the CCA is permanently ligated. In the repair group, a small tissue pad coated in fibrinogen and thrombin sealant was placed over the incision to produce a seal and allow full reperfusion of the CCA. At 5 min post-MCAO there was a significant increase in cerebral blood flow (CBF) in the ‘CCA repaired’ group (89.07±100.6%; *P*<0.001; Fig. 2) compared with CBF at the point of occlusion. There was a further significant increase at 5 min following the CCA repair and re-establishment of blood flow (140.1±142.5%; *P*<0.01; Fig. 2). This increase in reperfusion was specific to the repair of the CCA rather than a gradual increase over time as there was no significant difference between 5 min post-MCAO and the point immediately prior to the vessel being repaired (Fig. 2).

### Repairing the CCA reduces lesion volume and variability within groups

**Fig. 3.**
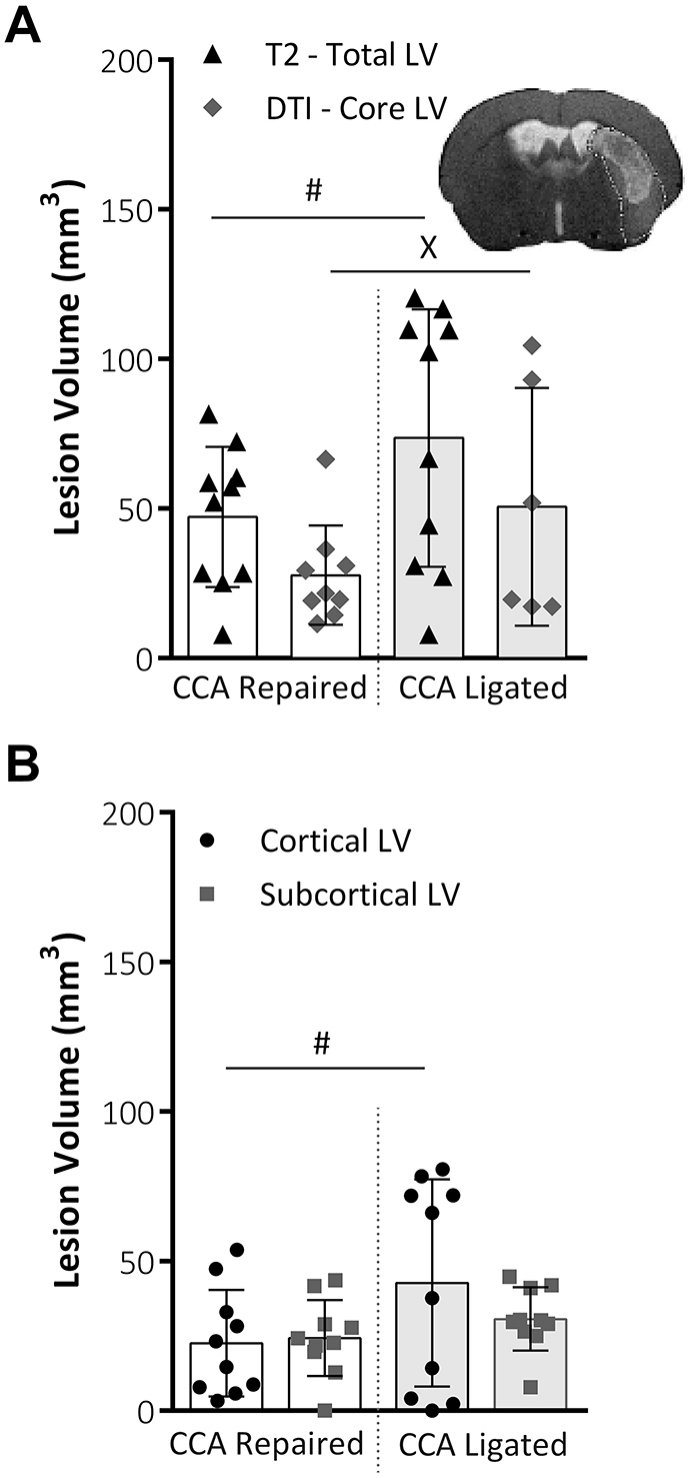
**MRI lesion volume analysis.** (A) Total lesion volume (mm^3^) at 48 h post-MCAO, as taken from T2-weighted MRI scans and core lesion volume as taken from DTI analysis (A). Variability within data, assessed using the *F*-test for parametric data or Lavene's test for non-parametric data, was significantly reduced for both total LV, i.e. T2-weighted scans, (*P*=0.015; CCA repair, *n*=10; CCA ligated, *n*=10; *F*-test) and core LV, i.e. DTI scans, (*P*=0.043; CCA repair, *n*=9; CCA ligated, *n*=6; Lavene's test). Representative image shows total lesion area from T2 scan slice with DTI core lesion volume mask overlaid. (B) Total lesion volume (mm^3^) at 48 h post-MCAO, taken from T2 MRI slices, split into cortical and subcortical lesion areas. CCA repair significantly reduced variability (*P*=0.03, *F*-test) in the cortical portion of lesion but did not affect variability in the sub-cortical portion of the lesion. Both groups, *n*=10. All data shown are mean±s.d., ^#^*P*<0.05, *F*-test; ^X^*P*<0.05, Lavene's test.

Animals in CCA repaired and CCA ligated groups underwent MRI to obtain total lesion volume from T2-weighted scans and core lesion volume from diffusion tensor MRI (DTI) scans (Fig. 3A). No significant differences were found between the two groups using total lesion volume (*P*=0.106) or core lesion volume (*P*=0.453). However, variability within the data groups was significantly reduced in the CCA repaired group for both total LV (T2-weighted scans: *P*=0.015; CCA repair *n*=10, CCA ligated *n*=10; *F*-test) and core LV (DTI scans: *P*=0.043; CCA repair *n*=9, CCA ligated *n*=6; Lavene's test). For T2w scans, it was possible to separate the lesion volume data into cortical and subcortical areas (Fig. 3B). CCA repair significantly reduced the variability in lesion volume (*P*=0.03; *F*-test) in the cortical portion of the lesion but did not affect variability in the sub-cortical portion of the lesion.

**Fig. 4.**
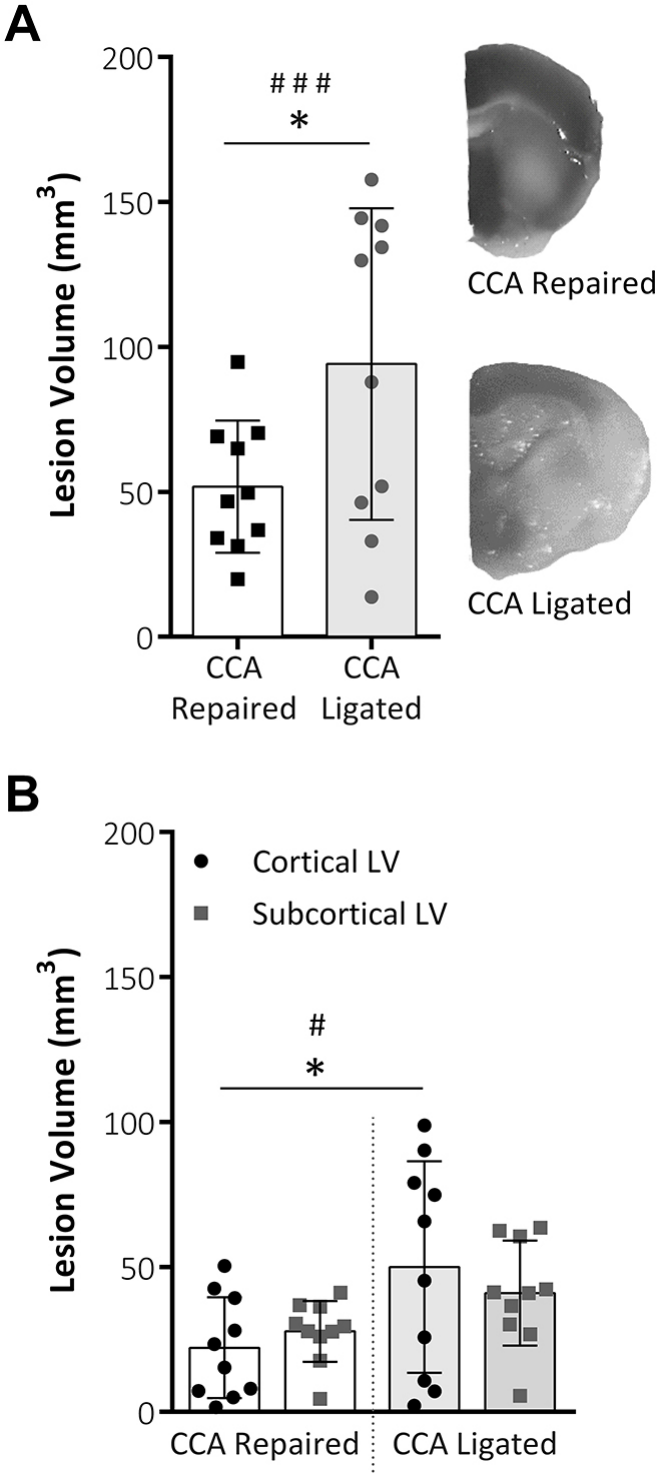
**TTC staining lesion volume analysis.** (A) Total lesion volume (mm^3^) at 48 h post-MCAO analysed from TTC-stained tissue slices. Both total lesion volume and data variability were reduced within the CCA repaired group. (B) Representative images of TTC staining of infarct area in each group. Total lesion volume split into cortical and subcortical lesion areas. TTC staining showed a reduced cortical component to the lesion following CCA repair compared with CCA ligation (*P*=0.0428); within the CCA repair group there was reduced data variability. Both groups, *n*=10; data are mean±s.d.; *P<0.05, ^#^*P*<0.05, F-test; ^###^*P*<0.01, F-test.

In addition to MRI analyses, lesion volume was also assessed using 2,3,5-triphenyltetrazolium chloride (TTC) staining, where white (i.e. unstained) areas depict ischemic tissue (Fig. 4A). Repairing the CCA significantly reduced the amount of lesion volume present (51.73±22.78 mm^3^) compared with the CCA ligated group (94.08±53.79 mm^3^; *P*=0.03). Variability was also significantly reduced in the CCA repaired group compared with the CCA ligated group (*P*=0.008; *F*-test). When the lesion was separated into cortical and subcortical areas in the CCA repaired group, there was a significant reduction in both the cortical lesion volume (22.10±17.4 mm^3^ vs 49.97±36.49 mm^3^, *P*<0.05) and the variability of the data (*P*<0.05; *F*-test) compared with results in the CCA ligated group (Fig. 4B).

**Fig. 5.**
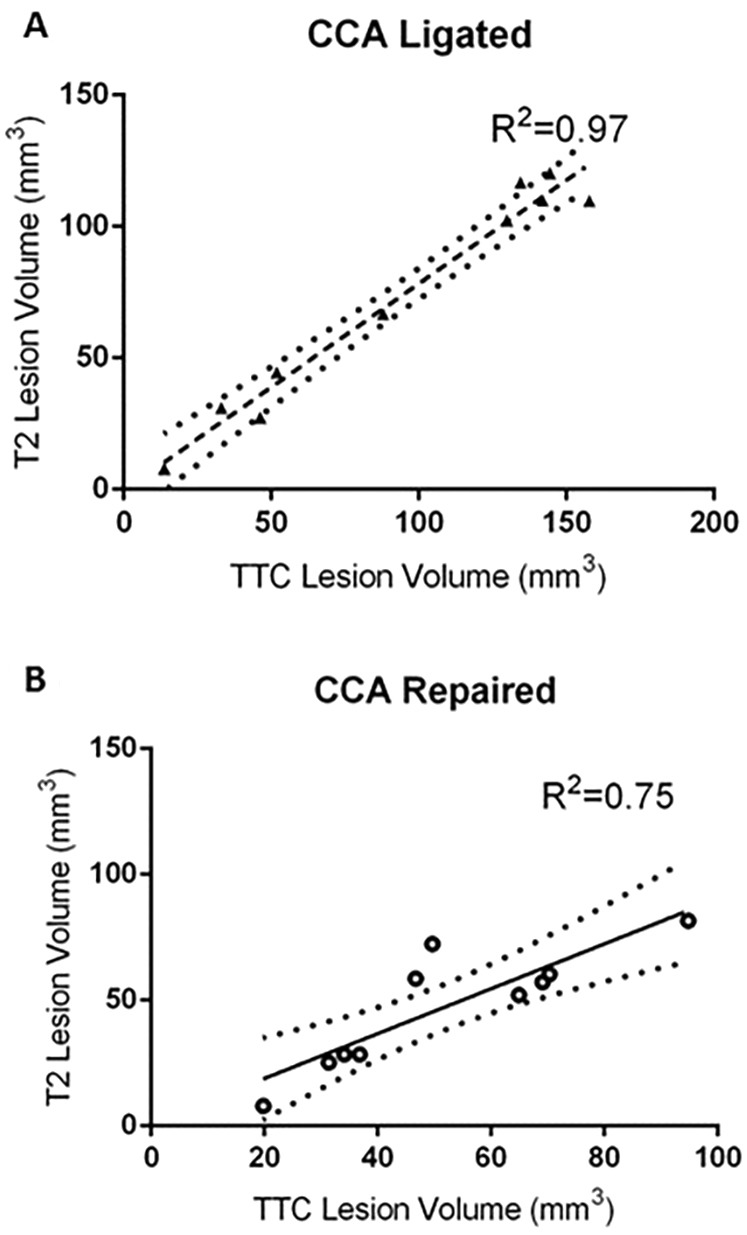
**Relationship between T2 lesion volume and TTC lesion volume.** (A) In the CCA ligated group, T2 and TTC lesion volume were positively correlated (*R*^2^=0.97, *P*<0.0001), slope of linear regression=0.79. (B) In the CCA repaired group, T2 and TTC lesion volume were also positively correlated (*R*^2^=0.75, *P*=0.0012), slope of linear regression=0.89. Dotted lines show 95% confidence band of the best fit line.

Lesion volume assessed by TTC staining and T2-weighted MRI were significantly correlated for both the CCA ligated and CCA repair groups (Fig. 5). The slope of the line of best fit was found to be lower in the CCA ligated group than the CCA repair group, indicating that T2-weighted MRI measured a systematically lower lesion volume than TTC staining, particularly in this group. This is supported by the lower mean total lesion volume measured by T2-weighted MRI versus TTC for the CCA ligated group (Fig. 3A versus Fig. 4A, 73.6 mm^3^ vs 94.1 mm^3^, respectively).

### Repairing the CCA increases the degree of ischemic injury damage within the lesion volume

**Fig. 6.**
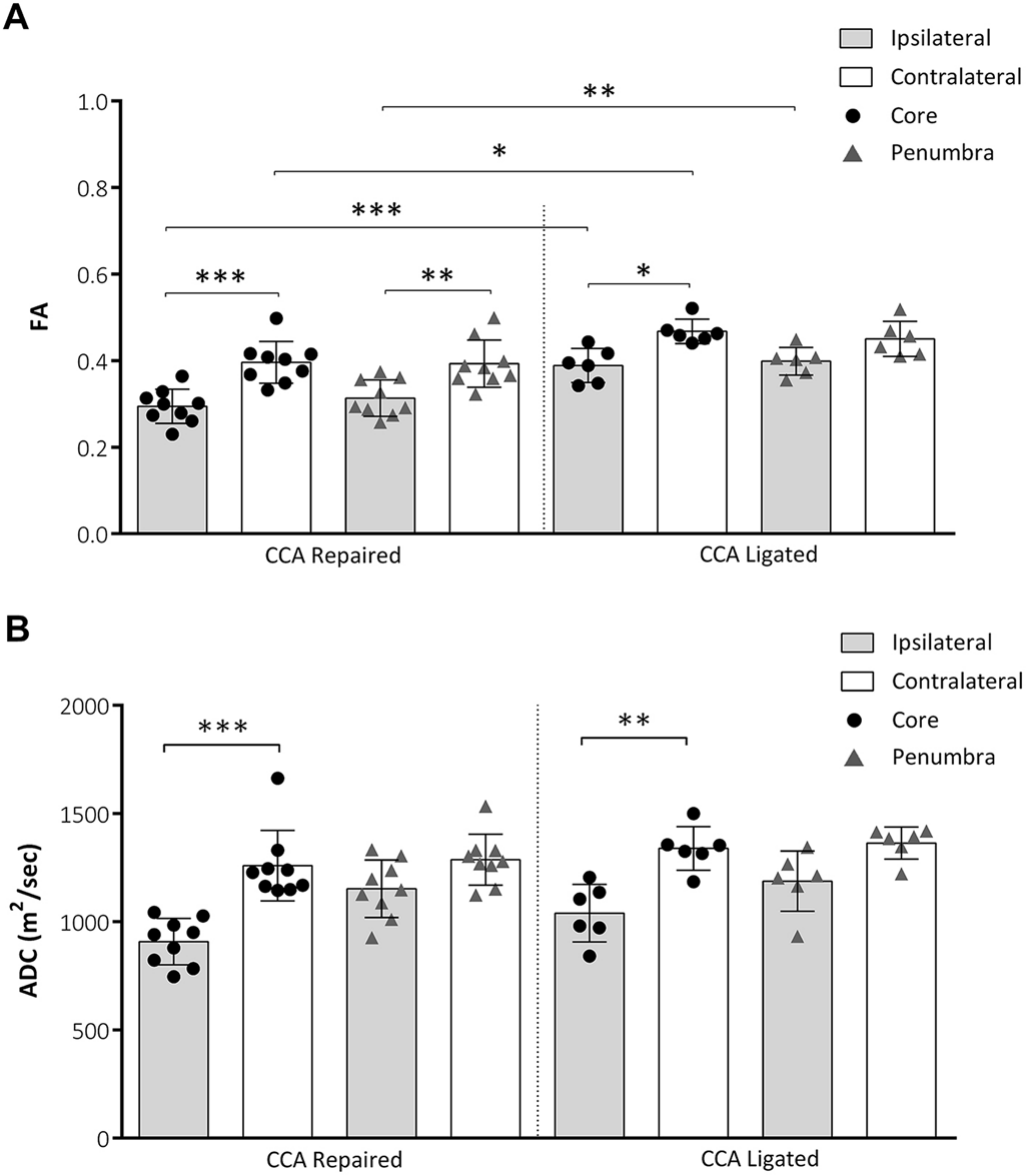
**Apparent diffusion coefficient (ADC) and fractional anisotropy (FA).** (A) FA at 48 h post-MCAO for core and penumbral ROIs in ipsilateral and mirrored contralateral hemispheres. FA was significantly lower in ipsilateral versus contralateral ROIs (CCA repaired: core and penumbra, CCA ligated: core only) and in repaired versus ligated ROIs (ipsilateral: core and penumbra, contralateral: core only). (B) ADC at 48 h post-MCAO for core and penumbral ROIs in ipsilateral and mirrored contralateral hemispheres. ADC was significantly lower in ipsilateral versus contralateral ROIs (CCA repaired and CCA ligated: core only). CCA repaired, *n*=9; CCA ligated, *n*=6. All data shown are mean±s.d., *P<0.05, **P<0.01, ***P<0.001.

Fractional anisotropy (FA) allows an estimate of local water diffusion anisotropy. In the current study, we measured FA for core and penumbral regions of interest (ROIs) in ipsilateral and mirrored contralateral hemispheres in the CCA repaired and CCA ligated groups. FA was significantly lower in the ipsilateral, i.e. ischemic, hemisphere compared with the contralateral hemisphere in the core region for both groups (CCA repaired, *P*<0.001; CCA ligated *P*=0.019) and in the penumbra region for the CCA repair group only (*P*=0.0019; Fig. 6B). The FA values were significantly lower in the ipsilateral, i.e. ischemic, hemisphere in the CCA repair group compared with the CCA ligated group for both the core (*P*=0.0008) and penumbral regions (*P*=0.0032).

Apparent diffusion coefficient (ADC) is a measure of the extent of water diffusion within tissue. In the current study, ADC values were significantly lower in the ipsilateral hemisphere compared with the mirrored contralateral hemisphere in the core region of the lesion for both the CCA repaired (*P*<0.001) and CCA ligated (*P*=0.0011) groups (Fig. 6B). For both groups, no significant difference was found in ADC values in the penumbral regions between the ipsilateral and contralateral hemispheres and no significant difference was found in ADC values between groups.

### Repairing the CCA reduces sample size needed to detect a possible treatment effect

From the data obtained in this study, a power analysis shows that fewer animals per group are required to demonstrate a 30% reduction in lesion volume following the CCA repaired method compared with the traditional CCA ligated approach (Table 1). Results are shown if we assume a power of 0.8, a significance level of 0.05 and predict a 30% reduction in lesion volume between the control group and ‘test’ group. Results are shown for the two approaches for inducing filament MCAO i.e. CCA ligated and CCA repaired to determine if there is a difference in the number of animals required. For each approach, equal variance is assumed between the control and test group.

**Table 1.**
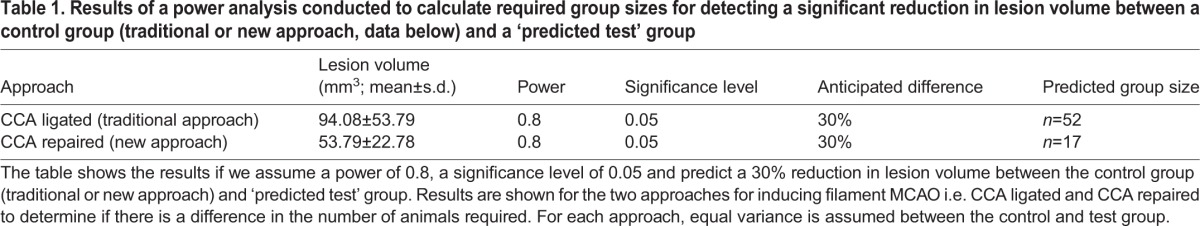
**Results of a power analysis conducted to calculate required group sizes for detecting a significant reduction in lesion volume between a control group (traditional or new approach, data below) and a ‘predicted test’ group**

## DISCUSSION

We report an alternative surgical approach for inducing filament MCAO in mice that results in reduced variability in lesion volume as assessed using a range of MRI analyses and histological staining. Avoiding ECA ligation and introducing an analgesic regime appears to reduce body weight loss without affecting lesion volume. Importantly, re-sealing the CCA reduced the variability of lesion volume along with changing the diffusion properties of the ischemic core.

Intraluminal MCAO is induced by inserting the filament via an incision at the trunk of the CCA, immediately below the bifurcation, or at the trunk of the ECA. ECA transection in rats can induce ischemic lesions of the mastication and swallowing muscles (Dittmar et al., 2003), affecting drinking behaviours and increasing body weight loss post-MCAO (Trueman et al., 2011). In mice, we show that when ECA ligation was avoided and an analgesic regime introduced there seemed to be no effect on lesion volume but data suggested that body weight loss was reduced following MCAO. However, owing to the experimental design it was not possible to separate out the possible effects of analgesia versus avoiding ECA ligation. Other reasons, including inter-individual animal variability and the failure to randomise within the same experiment, may also have contributed to the observed differences in body weight. A substantial number of experimental stroke studies do not report the use of analgesia due to possible confounding effects. However, there is a need to balance the appropriate and efficacious use of analgesics in rodents between the animal needs and the research objectives. In the current study, we used a combination of a non-steroidal anti-inflammatory drug (Carprieve) and an amide-type local anaesthetic (Marcaine), which did not appear to induce any neuroprotective effects.

The primary outcome measure in experimental stroke research tends to be infarct volume; however, large inconsistencies remain in ischemic lesion volume both within and between labs (Morris et al., 2016). Extensive research suggests that differences in animal strain, size, cerebrovascular anatomy, physiological variables, and size and type of filament all determine stroke outcome (Barone et al., 1993; Connolly et al., 1996). We predicted that, in mice, some of the variability, following CCA ligation, may be due to reliance on the Circle of Willis during reperfusion. Mice, in particular C57Bl/6 mice, have a highly anatomically variable Circle of Willis with 90% of animals having an incomplete Circle of Willis due to the lack of one or both posterior communicating arteries (PcomA; McColl et al., 2004). The patency of the PcomA is a crucial determinant of the extent of ischemic damage produced (Kitagawa et al., 1998). Repairing the CCA, as we describe here, enables blood flow to be re-established through the CCA and a previous study in rats showed that re-sealing the CCA did permit re-establishment of blood flow through the CCA (Dittmar et al., 2005). Although we did not specifically measure blood flow through the CCA, we did demonstrate that, following sealing of the CCA, reperfusion was successful and to a greater extent than seen in those animals in which the CCA was not repaired. We did not observe any failure in terms of the vessel not repairing, although longer-term studies would be required to rule out the possibility of the repair weakening over time. The method described here is technically challenging but requires no more technical skill than that required to surgically induce experimental stroke. Importantly, allowing the re-establishment of blood flow through the CCA reduced the variation seen in lesion volume when assessed by both TTC histology and imaging methods.

The main problem with a high variability in infarct size is the resulting lack of statistical power if the sample sizes are not adjusted accordingly. Statistical power in experimental stroke studies tends to be low; for example, in a recent meta-analysis (Ingberg et al., 2016), the average power of studies included was 59% to detect a 30% difference between groups (based on average group sizes of 8.4, average coefficient of variation for infarct size of 29.5% and a significance level of 0.05). Ethical requirements to use the minimum number of animals needed to achieve the scientific objectives along with economical and practical considerations undoubtedly contribute to studies being underpowered. However, if variability within groups can be reduced, as we have demonstrated in the current study, then this should help ensure that lesion sizes within groups are more consistent and appropriate *a priori* power calculations are performed. For example, using the data obtained in this study, our power analysis shows that fewer animals per group are required to demonstrate a 30% reduction in lesion volume following the CCA repaired method compared with the traditional CCA ligated approach.

Both the histological (i.e. TTC) and T2-weighted MRI data show a reduction in variability following repair of the CCA, verifying the main hypothesis of the study. A reduction in total and cortical lesion volume was found in the CCA repair group, significant for TTC data only, but with a similar trend in T2 data, and indicative of a more focal, subcortical and typically striatal lesion in the CCA repair group. Importantly, the reduction in lesion volume variability is most significant in the cortical region (Fig. 3B) and while variability in subcortical lesion volume also contributes to total lesion volume variability, the significant reduction in variability of the cortical component is most likely the largest contributing factor to the significant reduction in total lesion volume (Fig. 3A). Lesion volume assessed by TTC staining and T2-weighted MRI were significantly correlated for both the CCA ligated and CCA repair groups and it would seem that T2-weighted MRI resulted in a systematically lower lesion volume than TTC histology, particularly in the CCA ligated group. The exact mechanism behind this systematic difference is not clear. However, a significantly higher swelling correction factor for T2-weighted MRI versus TTC in the CCA ligated group is thought to be a contributing factor and the resultant reduction in mean lesion volume for the CCA repair group leads to an insignificant difference in lesion volumes between groups for T2-weighted MRI lesion volume data.

Information on the tissue alterations within the ischemic lesion was established by examining the diffusion-weighted MRI results. Both ADC and FA delivered discernible lesions at 48 h post-MCAO (Fig. S1) and were significantly lower in the ipsilateral region of interest compared with the contralateral region for both the CCA repaired and CCA ligated groups, indicating that decreased ADC and FA corresponds to degree of ischemic injury as suggested by previous human and animal studies (Barber et al., 2005; Hoehn et al., 2001; Ozsunar et al., 2004). This is supported by the relationship between lesion T2 hyperintensity and both ADC and FA (Fig. S2). While FA is known to increase initially following stroke, other studies have reported a decrease at 48 h post-MCAO (Ozsunar et al., 2004; Van der Zijden et al., 2008). Interestingly, FA was significantly lower in the core and penumbra region of the CCA repaired group compared with the CCA ligated group, suggesting that the degree of ischemic injury was higher within the lesions of the CCA repair group. Whilst a simultaneous reduction in both FA and ADC may appear contradictory, three temporally related phases have previously been described for the relationship between FA, ADC and stage of infarct: an acute phase during which anisotropy is increased and ADC is reduced, a second phase corresponding to a reduction in anisotropy and a reduction in ADC, and a third phase corresponding to a reduction in anisotropy and an elevation in ADC (Ozsunar et al., 2004; Yang et al., 1999). Considering our FA and ADC measurements were taken at 48 h post-MCAO it is reasonable to assume we are operating in the second phase where both FA and ADC are expected to be reduced.

In MRI studies, the standard method for separating the lesion core and penumbra is to use the so-called perfusion/diffusion mismatch approach (PWI; Heiss, 2010). In the absence of PWI data, as in the current study, it is feasible, as demonstrated in clinical stroke patients (Chen and Ni, 2012), to estimate total lesion volume from T2-weighted MRI and subtract the diffusion-weighted imaging (DWI) core from the T2 lesion to estimate penumbra volume. In clinical studies, a DWI scan is often taken within the first hours of stroke onset and T2 scans are acquired later, typically a number of days after stroke. The growth in the lesion between these two measurements is thought to estimate penumbra that is lost over the time between scans (Chen and Ni, 2012). In this study, with a single scan session at 48 h post-stroke, DWI and T2 scans were acquired at the same time point. Whilst this approach reproducibly leads to a penumbral ROI surrounding the DWI core (Movie 1), it should be viewed as a surrogate estimation of the ischemic penumbra, as it probably leads to an overestimation of penumbral volume by including oligemic effects and elevated T2 signal due to oedema (Ozsunar et al., 2004).

Combining the ADC and FA results with the reduction in lesion volume and lesion volume variability described above, we can conclude that the CCA repair method provides a more focal, less variable lesion volume but with a higher degree of ischemic tissue damage. The method can therefore provide a more suitable baseline for future drug treatment studies. In conclusion, this study shows that an alternative surgical approach is feasible for inducing MCAO in mice whereby welfare is improved and variability within groups decreased. The importance of this work is in demonstrating that reduced variability in animal stroke studies has the potential to increase animal wellbeing, reduce the number of animals used and potentially increase the efficacy of animal studies in detecting treatment effects.

## MATERIALS AND METHODS

### Animals

This study was conducted in accordance with the UK Animals (Scientific Procedures) Act, 1986 (Project license 60/4315) and following institutional ethical approval. All mice were adult male C57 Bl6\j (Charles River, Oxford, UK) weighing between 24 and 31 g at the time of surgery. A total of 24 male mice were used in the current study: one died following middle cerebral artery occlusion (MCAO; Fig. S3) and one was excluded because of surgical complications. All experiments are reported in accordance with the Animal research: reporting of *in vivo* experiments (ARRIVE) guidelines.

### Focal cerebral ischemia

All mice underwent focal cerebral ischemia by transient MCAO as previously described (Gibson and Murphy, 2004), with specific modifications as detailed below (see also Fig. S4). Mice were randomly assigned to either the CCA ligated or CCA repaired group and the surgeon was masked to all subsequent analyses. Mice were anaesthetised with isoflurane (induction 5%; maintenance 1.5% in N_2_O/O_2_, 70/30%). Body temperature was monitored during surgery (via a rectal probe) and maintained at 37.0±0.6°C using a heating blanket (Harvard Apparatus). In order to induce MCAO, a midline incision was made on the ventral surface of the neck, and the right CCA isolated and ligated with a 6.0 silk suture. Unlike previous studies using this model (for example, see Gibson et al., 2014; Trotman et al., 2015), ligation of the ECA was avoided. The CCA was temporarily occluded using a microvascular clip (WPI Ltd., Europe). A nylon monofilament (Doccol, USA; filament size 7-0, coating diameter 0.19±0.01 mm, coating length 5-6 mm) was introduced into the intracranial internal carotid artery through an incision made in the CCA. The filament was advanced to the carotid bifurcation in order to occlude blood flow into the middle cerebral artery. Laser Doppler flowmetry (Moor Instruments) was used to monitor cerebral blood flow for 5 min before and 5 min after MCAO, and immediately before and 5 min after reperfusion. A small incision was made in the skin overlying the temporalis muscle and a 0.7 mm flexible Laser Doppler probe (model P10) was positioned on the superior portion of the temporal bone (6 mm lateral and 2 mm posterior from Bregma) secured with Superglue (Loctite). Once successful occlusion had been determined, animals were recovered in the home cage on paper 3Rs bedding. Following 60 min of MCAO, animals were re-anaesthetised and the filament withdrawn in order to allow reperfusion to take place. In the CCA ligated group, the standard procedure was followed whereby the filament was removed from the vessel and the sutures tied in place on the CCA to prevent blood loss through the incision. In the CCA repair group, the filament was removed and a small tissue pad taken from surrounding tissue was used in combination with TISEEL (Baxter Healthcare) to seal the incision in the CCA. Thus, in the CCA repair group re-establishment of blood flow through the CCA was permitted.

### Analgesia and welfare monitoring

All mice received Carprieve (10 mg/kg, 5% w/v, Norbrook Laboratories Ltd., UK) injected subcutaneously immediately prior to undergoing MCAO surgery along with Marcaine (2 mg/kg, 0.25% w/v, AstraZeneca, UK) applied directly to the skin surrounding the wound site at the point of wound suture. Following MCAO, grimace scoring (Langford et al., 2010) was used to assess post-operative pain levels post-MCAO in order to aid the decision as to whether further analgesia was required. In the current study, no additional analgesia was administered to any mice. Mice were weighed at 24 and 48 h post-MCAO as an indicator of their general well being and body weight changes presented as a percentage change compared with pre-MCAO levels. For each mouse, daily observation score sheets were completed to record the presence of any clinical signs. All mice received subcutaneous 200 µl injections of pre-warmed saline twice daily following MCAO and had unrestricted access to soggy pellets, wet mashed pellets, diet gel (76A Purified soft diet, ClearH2O.com), hydrogel (98% sterile water, ClearH2O.com), dry food pellets and water. At 24 and 48 h post-MCAO, mice were assessed on a general deficit scale (evaluating hair, eyes, posture, spontaneous activity and any signs of epileptic behaviour) and a focal deficit scale (evaluating body symmetry, gait, climbing a 45° barred surface, circling behaviour, front limb symmetry, compulsory circling and whisker responses to touch) – both scales range from 0 (normal) to 28 (Clark et al., 1998; De Simoni et al., 2003; Orsini et al., 2010). Survival rates of the two experimental groups (i.e. CCA ligation versus CCA repair) are also presented as a percentage of the number of animals undergoing surgery (Fig. S1).

### MRI and image processing

MRI scanning was performed on a 9.4T Agilent scanner (Agilent Technologies, Santa Clara, CA, USA) with a 310 mm bore diameter and 12 cm inner-diameter gradient (600 mT/m maximum gradient strength), interfaced with a DirectDrive Console. DTI and T2-weighted MRI images were acquired at 48 h post-MCAO. Infarct volumes were delineated on T2-weighted images. ADC and FA maps were generated from DTI data. Mice were fixed in a mouse holder with a bite bar and ear inserts (RAPID Biomedical, Rimpar, Germany). Physiological monitoring was achieved using a custom monitoring and gating system (SA Instruments, Stony Brook, NY, USA). Body temperature was maintained at 37°C using a warm air fan and rectal temperature probe. Respiration was measured using a pneumatic pillow. All scans were performed during the light cycle under anaesthesia (1-2% isoflurane) in oxygen. Radio frequency transmission and reception was achieved with a 72 mm volume coil and 2-channel surface receiver coil specifically for mouse brain imaging, respectively (RAPID Biomedical). The mouse brain was positioned at the isocenter of the magnet and located with fast gradient echo scan. 3D gradient echo shimming of first- and second-order shims was performed on a whole-brain voxel and shim quality was confirmed using point resolved spectroscopy (PRESS) of the water peak. T2-weighted images were acquired using a fast spin echo (FSE) sequence with TR/TE=3000/40 ms, 18×18 mm field of view (256×256 matrix), 18×0.8 mm coronal slices and three signal averages (scan duration=9 min 42 s). DTI images were acquired using a FSE sequence with TR/TE=1730/35 ms, 20×20 mm field of view (128×128 matrix), 16×1.0 mm coronal slices, two signal averages, 14 encoding directions, 10.37 G/cm gradient pulse of 5 ms duration and 26 ms separation, maximum b-value=1024 s/mm^2^ (scan duration=13 min). All images were corrected for intensity inhomogeneities introduced by the 2-channel surface receiver coil using the bias field correction method in 3DSlicer, Version 3.6 (http://www.slicer.org; Fedorov et al., 2012). ROI analysis of lesion volume was performed on T2-weighted images in ImageJ Fiji (www.imagej.net; Schindelin et al., 2012). Image slices including a lesion component were identified and three repeat measurements of lesion area, ipsilateral hemisphere area and contralateral hemisphere area were manually delineated on each slice by an experienced operator, blinded to experimental group allocation. Area measurements were multiplied by slice thickness to calculate volume. Total lesion volume was corrected for the effects of edema by dividing lesion volume by the ratio of ipsilateral to contralateral hemisphere volume on a slice-by-slice basis.

ADC and FA maps were generated from DTI data using the diffusion analysis method in VnmrJ, v.4.2 (Agilent Technologies, Santa Clara, CA, USA). Lesion core ROIs were identified on thresholded diffusion-weighted images. Linear registration of diffusion-weighted and T2-weighted images was performed using the General registration (BRAINS) module in 3DSlicer. Following registration, subtraction of diffusion-weighted lesion core ROIs from T2-weighted total lesion ROIs resulted in an estimated penumbra ROI, as illustrated in Fig. S3C. These ROIs were applied to ADC and FA maps to calculate mean ADC and FA within the core and penumbra.

### Lesion volume

At 48 h post-MCAO and immediately after the end of the MRI scanning, animals were sacrificed using cervical dislocation. Removed brains were sectioned, stained using 2% 2,3,5-triphenyltetrazolium chloride (TTC; Sigma) in saline, fixed and imaged to allow for quantification of ischemic damage. Brains were sectioned into 10×1 mm coronal slices using a mouse brain matrix (ASI Instruments). To allow for quantification of ischemic damage, slices were stained with 2% TTC in saline for 30 min at room temperature in the dark. They were then stored at 4°C in 10% formalin prior to analysis. TTC is a marker of mitochondrial function and an indicator of ischemic damage. Digital photographs of all stained slices were taken and the area of infarct calculated using Scion Image whereby overestimation of the infarct area due to edema is avoided (Loihl et al., 1999).

### Statistical analysis

All data are expressed as mean±s.d. The data were analysed using GraphPad Prism v.6.05 for Windows (GraphPad). The criterion for statistical significance was *P*<0.05. Data was first examined to assess distribution using the D'Agostino and Pearson omnibus normality test. Parametric data comparing two means (TTC and T2 MRI lesion volume data) were compared using Student's *t*-test. Parametric data comparing more than two means was compared using one-way ANOVA (cerebral blood flow, FA/ADC data) with Šidák test for multiple comparisons. Non-parametric data comparing two means (DTI lesion volume) were compared using the Mann–Whitney test. Variability of data was assessed using the *F*-test for parametric data (TTC, T2-weighted MRI scans) or Lavene's test for non-parametric data (DTI scans).
