# Correction: An alternative surgical approach reduces variability following filament induction of experimental stroke in mice (doi: 10.1242/dmm.029108)

**DOI:** 10.1242/dmm.033407

**Published:** 2018-01-01

**Authors:** Melissa Trotman-Lucas, Michael E. Kelly, Justyna Janus, Robert Fern, Claire L. Gibson

Errors were published in a table in *Dis. Model. Mech.* (2017). **10**, 931-938 (doi: 10.1242/dmm.029108).

The corrected table (Table 1) is below and the original article has been changed correspondingly.

**Table 1.**
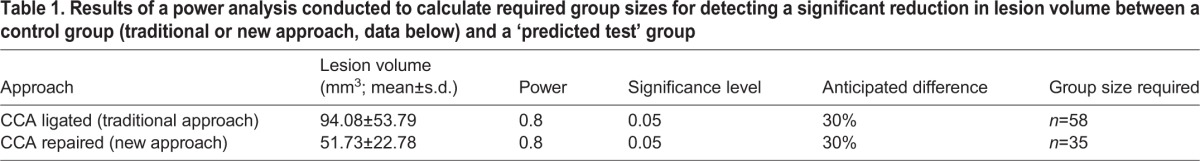
**Results of a power analysis conducted to calculate required group sizes for detecting a significant reduction in lesion volume between a control group (traditional or new approach, data below) and a ‘predicted test’ group**

The table shows the results if we assume a power of 0.8, a significance level of 0.05 and predict a 30% reduction in lesion volume between the control group (traditional or new approach) and ‘predicted test’ group. Results are shown for the two approaches for inducing filament MCAO i.e. CCA ligated and CCA repaired to determine if there is a difference in the number of animals required. For each approach, equal variance is assumed between the control and test group.

